# Vesicle-associated Membrane Protein 2 (VAMP2) but Not VAMP3 Mediates cAMP-stimulated Trafficking of the Renal Na^+^-K^+^-2Cl^−^ Co-transporter NKCC2 in Thick Ascending Limbs*

**DOI:** 10.1074/jbc.M114.589333

**Published:** 2014-07-09

**Authors:** Paulo S. Caceres, Mariela Mendez, Pablo A. Ortiz

**Affiliations:** From the ‡ Hypertension and Vascular Research Division, Department of Internal Medicine, Henry Ford Hospital, Detroit, Michigan 48202 and; the § Department of Physiology, Wayne State University, Detroit, Michigan 48202

**Keywords:** Cyclic AMP (cAMP), Epithelial Cell, Exocytosis, Kidney, Membrane Trafficking, SNARE Proteins, Na-K-Cl Co-transporter 2 (NKCC2), Epithelial Sodium Transport

## Abstract

In the kidney, epithelial cells of the thick ascending limb (TAL) reabsorb NaCl via the apical Na^+^/K^+^/2Cl^−^ co-transporter NKCC2. Steady-state surface NKCC2 levels in the apical membrane are maintained by a balance between exocytic delivery, endocytosis, and recycling. cAMP is the second messenger of hormones that enhance NaCl absorption. cAMP stimulates NKCC2 exocytic delivery via protein kinase A (PKA), increasing steady-state surface NKCC2. However, the molecular mechanism involved has not been studied. We found that several members of the SNARE family of membrane fusion proteins are expressed in TALs. Here we report that NKCC2 co-immunoprecipitates with VAMP2 in rat TALs, and they co-localize in discrete domains at the apical surface. cAMP stimulation enhanced VAMP2 exocytic delivery to the plasma membrane of renal cells, and stimulation of PKA enhanced VAMP2-NKCC2 co-immunoprecipitation in TALs. *In vivo* silencing of *VAMP2* but not *VAMP3* in TALs blunted cAMP-stimulated steady-state surface NKCC2 expression and completely blocked cAMP-stimulated NKCC2 exocytic delivery. VAMP2 was not involved in constitutive NKCC2 delivery. We concluded that VAMP2 but not VAMP3 selectively mediates cAMP-stimulated NKCC2 exocytic delivery and surface expression in TALs. We also demonstrated that cAMP stimulation enhances VAMP2 exocytosis and promotes VAMP2 interaction with NKCC2.

## Teaser

**Background:** Exocytic delivery of the renal co-transporter NKCC2 to the cell surface is a major mechanism for NaCl reabsorption.

**Results:** We describe a mechanism that mediates cAMP-stimulated NKCC2 delivery in renal cells.

**Conclusion:** Vesicle fusion protein VAMP2 interacts with NKCC2 and mediates cAMP-stimulated NKCC2 exocytic delivery.

**Significance:** The molecular mechanism mediating NKCC2 exocytic delivery could provide new targets for treatment of hypertension.

## Introduction

NaCl reabsorption by the thick ascending limb (TAL)^2^ is a fundamental process for creating a hyperosmotic renal medulla, which provides the driving force for concentrating urine. Approximately 100% of the chloride and 50% of the sodium absorbed in this nephron segment are transported across the apical membrane via the Na^+^/K^+^/2Cl^−^ co-transporter NKCC2. Under steady-state conditions, a small fraction of NKCC2 (∼5%) is present at the apical membrane, with most of the remaining transporter located in an intracellular subapical pool (1–3). There is a close relationship between the presence of active co-transporters at the cell surface and the capacity of the TAL to absorb NaCl (3–8). Maintenance of steady-state surface NKCC2 levels is a dynamic process resulting from the balance between exocytic delivery (9), endocytic retrieval, and recycling (8, 10). Biological pathways that stimulate cAMP production, such as hormonal stimulation with arginine vasopressin or β-adrenergic receptors, enhance NaCl reabsorption by the TAL by increasing steady-state surface NKCC2 expression (3–5, 9). In a similar way, factors that decrease NaCl transport, including nitric oxide and cGMP, decrease the abundance of NKCC2 at the apical surface of the TAL (7).

It is accepted that an increase in steady-state surface protein levels can be achieved in two ways: (i) by stimulating the rate of exocytosis or (ii) by decreasing endocytic retrieval from the plasma membrane, thereby causing the protein to accumulate at the surface. In the TAL, surface NKCC2 is maintained by constitutive exocytic delivery (9), endocytic retrieval, and recycling that occurs in the absence of hormonal stimuli (8, 10). However, when cAMP is elevated, NKCC2 exocytic delivery is increased via protein kinase A (PKA), resulting in higher surface NKCC2 levels (9). Information regarding the molecular components and proteins involved in cAMP-stimulated NKCC2 delivery to the apical membrane is practically nonexistent.

In all other cells studied to date, exocytosis is achieved via assembly of a SNARE (soluble NSF attachment protein receptor) complex composed of three types of proteins: vesicle-associated membrane proteins (VAMPs), synaptosome-associated proteins (SNAPs), and syntaxins (11, 12). One VAMP isoform present in the vesicle membrane recognizes the other two SNAREs, SNAP and syntaxin, in the target membrane (13, 14). This structure is the SNARE complex and is the first intermediate driving the membrane fusion process (15, 16). Little is known about the role and function of SNARE proteins in mediating apical trafficking in renal epithelia. Few SNARE proteins have been reported in the kidney (3, 17–20), and their role in NKCC2 trafficking is unknown. We previously found that VAMP2 and VAMP3 are present in the apical membrane and subapical space of TALs (3). In addition, treatment of TALs with tetanus toxin, which cleaves both VAMP2 and VAMP3, blocked the stimulatory effect of cAMP on NKCC2-mediated NaCl absorption by the TAL (3), suggesting a potential role of VAMPs in stimulated NKCC2 trafficking. In non-renal cells, VAMP2 has been associated with stimulated exocytosis events (21–24). In addition, VAMP2 is known to mediate cAMP-stimulated exocytosis in nerve cells (25) and in renal cells of the juxtaglomerular apparatus (26). However, it is unclear whether VAMP2 also mediates cAMP-stimulated NKCC2 exocytic insertion in the TAL or which are the mechanisms involved. We tested the hypothesis that VAMP2 mediates all of the cAMP-stimulated exocytic insertion of NKCC2 required for enhancing steady-state surface NKCC2 levels in freshly isolated TALs.

## EXPERIMENTAL PROCEDURES

### Drugs and Antibodies

Reagents for steady-state surface biotinylation and exocytic insertion protocols were obtained from Thermo Fisher Scientific Inc. (Waltham, MA), forskolin and IBMX were from Sigma, and *N*^6^-benzoyl-cAMP was from Biolog (Bremen, Germany). The chicken anti-NKCC2 antibody recognizes amino acids 33–55 of the amino terminus of rat NKCC2, the same sequence reported for the L-320 antibody by Kim *et al.* (27). The rabbit anti-NKCC2 antibody was produced by GenScript (Piscataway, NJ) and recognizes amino acids 859–873 in the carboxyl terminus of rat NKCC2. The monoclonal anti-GAPDH antibody was from Chemicon (Temecula, CA). The mouse antibody against VAMP2 and the rabbit antibodies against VAMP8, SNAP-23/29/47, and syntaxins 3/4/7 were from Synaptic Systems (Goettingen, Germany). The rabbit anti-VAMP3 and the mouse anti-VAMP7 antibodies were from Abcam (Cambridge, MA). The mouse anti-SNAP-25 antibody was from Covance (Princeton, NJ). The anti-phosphoserine 126 NKCC2 antibody was generously provided by Dr. Mark Knepper (28). The mouse anti-GFP antibody was from Clontech (Mountain View, CA). The rabbit Alexa Fluor 488-conjugated anti-GFP and anti-rabbit Alexa Fluor 568-conjugated antibodies were from Invitrogen. The HRP-conjugated secondary anti-mouse and anti-rabbit antibodies were obtained from GE Healthcare, HRP-conjugated secondary anti-chicken IgG was from Gallus Immunotech (Fergus, Canada), and rabbit non-immune IgG was from Sigma.

### Primary Culture of TALs

Individual TAL cells were obtained from rat TAL suspensions after digesting the tubules with collagenase (0.1%), trypsin (0.25%), and DNase (0.0021%). Single TAL cells were isolated by centrifugation at 41,300 × *g* in a 35% Percoll gradient and seeded in permeable supports (Corning Inc.) coated with basement membrane extract (Trevigen, Gaithersburg, MD). Cells were grown in DMEM supplemented with 1% fetal bovine serum and insulin-transferrin-selenium supplement (Invitrogen) at 37 °C and 5% CO_2_.

### Immunolabeling of Surface NKCC2 and Surface VAMP2 in TAL Cells

Cultured TAL cells were transduced with adenoviruses expressing VAMP2-eGFP under control of the CMV promoter for 24 h. The VAMP2-eGFP construct, generously provided by Dr. Regazzi (29), was subcloned into a VQ Ad5CMV K-NpA adenovector (Viraquest). Viral particles were produced and purified by Viraquest. TAL cells were treated with either vehicle or forskolin (20 μm) plus IBMX (0.5 mm) for 30 min at 37 °C. Protein trafficking was rapidly stopped by adding cold medium and incubating the cultures at 4 °C for 30 min. Cells were blocked with 5% albumin in physiological solution for 30 min. Then a primary rabbit antibody against an exofacial epitope on NKCC2 (7–9) was added to the apical bath (1:100) at 4 °C for 2 h. Cells were washed and incubated with the secondary anti-rabbit antibody conjugated with Alexa Fluor 568 (1:100) for 1 h at 4 °C. After washout, surface VAMP2-eGFP was labeled with Alexa Fluor 488-conjugated anti-GFP (1:100) for 1 h at 4 °C. Finally cells were fixed in 4% paraformaldehyde and mounted. Images were acquired using a laser-scanning confocal microscopy system (Visitech, Sutherland, UK) with a 488-nm diode or krypton/argon 568-nm gas laser excitation controlled by an acousto-optic tunable filter. Images were acquired at ×100 (1.4 numerical aperture), and fluorescence was observed using 525/55-nm BP or 590-nm LP filters, respectively. Image files (TIFF) were minimally deconvolved with Autoquant software (Media Cybernetics) using two-dimensional blind deconvolution. Images from both channels were aligned, and pixel-by-pixel co-localization was measured using a minimum Mander's overlap coefficient of 0.95. An image for overlapping pixels was generated.

### In Vivo Gene Silencing

All protocols were approved and conducted in accord with Institutional Animal Care and Use Committee guidelines.

### Construction of Adenoviral Vectors

The target sequences for VAMP2 and VAMP3 silencing were 19 nucleotides from the rat genes: *VAMP2* (5′-GCTCAAGCGCAAATACTGG-3′) and *VAMP3* (5′-GGATCTTCTTCGAGACTTT-3′), which were introduced into adenoviruses as described previously (26). Nude small interfering RNAs (siRNAs) were synthesized with the Silencer siRNA construction kit from Applied Biosystems (Carlsbad, CA) and tested *in vitro* in NRK-52E cells from the American Type Culture Collection (Manassas, VA). Sense and antisense sequences spaced by a loop sequence (TTCAAGAGA) were subcloned between the 5′ AflII and 3′ SpeI sites in the Adenovector-pMIGHTY (Viraquest, North Liberty, IA) to produce adenoviral particles coding short hairpin RNAs (shRNAs) for *VAMP2* and *VAMP3*. The constructs were sequenced, and replication-deficient adenoviral particles were assembled by Viraquest. A control construct coding a non-targeting sequence (5′-TTCTCCGAACGTGTCACGT-3′) was produced in the same way. The adenoviruses were tested again in NRK-52E cells at 100 plaque-forming units/cell. Expression of VAMP2, VAMP3, VAMP7, VAMP8, and GAPDH was monitored by Western blot.

### In Vivo Transduction of TALs

Male Sprague-Dawley rats weighing 130 g (Charles River Breeding Laboratories, Wilmington, MA) were anesthetized with ketamine and xylazine, and adenoviruses carrying shRNA sequences for *VAMP2* or control were injected into the outer medulla as described previously (10, 30, 31). A small incision was made in the left flank to expose the kidney. The renal artery and vein were clamped to stop blood flow and prevent washout of adenoviruses. Five 20-μl injections, 1 min each, were performed with a premeasured 30-gauge needle connected to a nanoliter syringe pump at 20 μl/min. After 8 min, the clamp was released to reestablish renal blood flow. The kidney was returned to the abdominal cavity, and the incision was closed. The procedure was then repeated in the right kidney of the same rat but using the control shRNA sequence.

### Steady-state Surface Biotinylation of NKCC2 in TAL Suspensions

Biotinylation of TAL surface NKCC2 was performed as described before in detail (3, 7). TALs were equilibrated at 37 °C for 15 min and then treated with either vehicle or forskolin (20 μm) plus IBMX (0.5 mm) for 30 min. TALs were biotinylated at 4 °C with NHS-SS-biotin (0.9 mg/ml), washed, and lysed in buffer containing 150 mm NaCl, 50 mm HEPES (pH 7.5), 5 mm EDTA, 1% Triton X-100, 0.2% SDS, and protease inhibitors. Biotinylated proteins were separated with streptavidin-coated beads overnight at 4 °C and recovered by boiling in Laemmli loading buffer containing dithiothreitol (DTT) and β-mercaptoethanol. Proteins were resolved in 6% SDS-polyacrylamide gels, and NKCC2 and GAPDH were detected by Western blot. In every experiment, we monitored total NKCC2 expression and absence of intracellular biotinylation by examination of GAPDH expression in the surface fraction. Bands were detected by chemiluminescence and quantified.

### Exocytic Delivery of Surface Proteins

#### 

##### NKCC2 Exocytic Delivery

Surface proteins accessible to NHS-SS-biotin in TAL suspensions were first masked by reaction with membrane-impermeant NHS-acetate as described previously (9). Briefly, TALs were incubated with 2 mg/ml NHS-acetate at pH 7.8 and 4 °C for 1 h, adding fresh NHS-acetate every 15 min. Forskolin and IBMX were added at 4 °C, and samples were warmed to 37 °C for 30 min. TALs were then cooled, and newly inserted NKCC2 was biotinylated with NHS-SS-biotin. The efficiency of NHS-acetate masking for NKCC2 was calculated in every experiment from the difference between a TAL aliquot that was not masked with NHS-acetate (100% basal surface NKCC2) and an aliquot that was masked at 4 °C but never warmed to 37 °C (0 time point). The difference between these two samples represents the NHS-acetate-masked surface NKCC2 fraction, which was used to express exocytic insertion over time.

##### VAMP2-eGFP Exocytic Delivery

Primary cultures of TALs were obtained from rats and transfected with VAMP2-eGFP via adenoviruses. The VAMP2-eGFP construct was provided by Dr. Regazzi (University of Lausanne, Switzerland) (29). Twenty-four hours after transfection, cells were treated with either vehicle or forskolin (20 μm) plus IBMX (0.5 mm). The GFP portion of VAMP2-eGFP is carboxyl-terminal and faces the extracellular space, being accessible for biotinylation. We measured exocytic delivery by masking with NHS-acetate and biotinylating newly delivered VAMP2-eGFP in the same way as described above for NKCC2. Surface VAMP2-eGFP was measured by Western blot with anti-GFP antibody.

### Total Internal Reflection Fluorescence (TIRF) Imaging of VAMP2 Exocytosis

Epithelial LLC-PK1 cells were grown in glass coverslips and transfected with VAMP2-eGFP. Cells were transferred to a temperature-controlled chamber maintained at 37 °C and imaged by TIRF microscopy (Nikon TE2000U, equipped with “through the lens” TIRF module, ×100, 1.45 numerical aperture lens). After selecting a group of cells expressing VAMP2-eGFP, the 488-nm excitation laser was angled until reflection was observed. Images were acquired at 1024 × 1024 resolution, every 500 ms for a 10-min control period. Then forskolin and IBMX were added to the chamber, and images were acquired for an additional 10 min and stored. Stored images were analyzed with Metafluor software (Molecular Devices, Sunnyvale, CA). Regions of interest were defined at locations where exocytic events were observed, and fluorescence intensity profiles were obtained. Exocytic events were defined as transient increases in total fluorescence that lasted less than 20 s and were higher in intensity than 3 times the S.D. value from a 25-point moving average.

### Co-Immunoprecipitation

TAL protein samples were obtained from suspensions lysed in buffer containing 150 mm NaCl, 50 mm HEPES (pH 7.5), 5 mm EDTA, 1% Triton X-100, 0.1% SDS, proteases, and phosphatase inhibitors from Roche Applied Science. 200 μg of protein from TALs were precleared for 30 min at 4 °C with protein G/protein A-coupled agarose beads (Thermo Fisher Scientific) in immunoprecipitation buffer containing 100 mm NaCl, 50 mm HEPES (pH 7.5), 5 mm EDTA, 1% Triton X-100, 1% CHAPS, and protease inhibitors. Beads were precipitated by centrifugation and discarded. Supernatants were incubated with 5 μg of rabbit anti-NKCC2 IgG (27) overnight at 4 °C. Control tubes were incubated with a non-immune rabbit IgG. The next day, protein G/protein A-coupled agarose beads were added in two sequential rounds for a total of 4 h at 4 °C. At the end of the incubation period, beads were sequentially washed in immunoprecipitation buffer, in 500 mm sodium/50 mm HEPES buffer, and finally in no-sodium/50 mm HEPES buffer. Proteins were extracted by incubation at 37 °C in loading buffer and loaded in 5% stacking, 12% resolving SDS-polyacrylamide gels. Proteins were transferred to PVDF membranes and blocked in 5% milk in TBS-T buffer for 1 h at room temperature. Next, membranes were blotted with either primary monoclonal anti-VAMP2 or VAMP7 or polyclonal antibody anti-VAMP8 at 1:2000, 1:500, and 1:5000 dilutions, respectively, for 1 h at room temperature. Finally, membranes were incubated with the corresponding HRP-conjugated secondary antibody at 1:5000 for 1 h at room temperature and developed by chemiluminescence. For VAMP2 immunoprecipitation, we used 100 μg of protein lysate and 5 μg of anti-VAMP2 or non-immune rabbit IgG. 6% SDS-polyacrylamide gels were run, and NKCC2 was blotted as indicated for surface biotinylation.

### Statistics

Results are expressed as mean ± S.E. One-way analysis of variance was used to determine differences between means in treatments with more than two groups, and Student's *t* test was used for comparisons between two groups. *p* < 0.05 was considered significant.

## RESULTS

### 

#### 

##### Expression of SNARE Proteins in Thick Ascending Limbs

**FIGURE 1.**
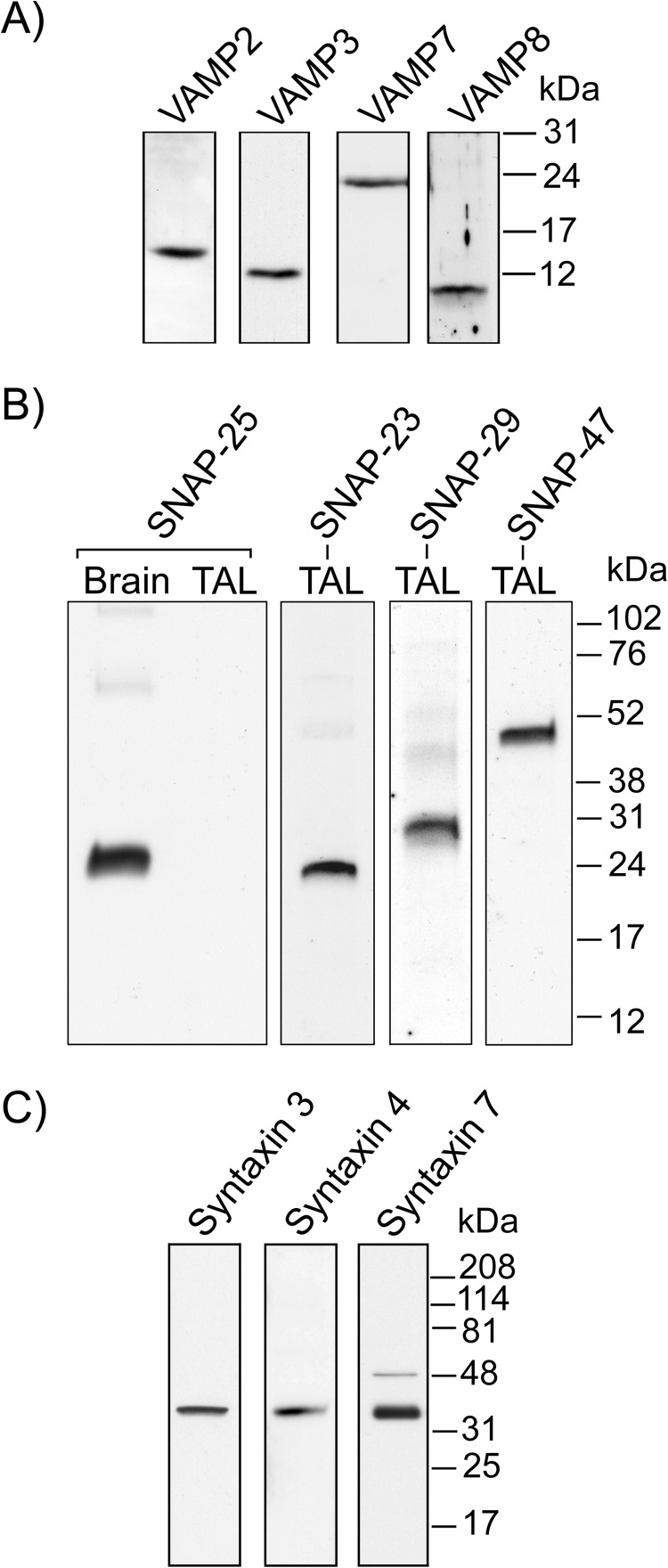
**Expression of VAMPs and target membrane SNAREs in the TAL.** *A*, VAMP isoforms 2, 3, 7, and 8 were found in isolated TALs (*n* = 4). *B*, SNAP isoforms 23, 29, and 47 are expressed in isolated TALs. SNAP-25, although highly expressed in brain tissue, was not found in the TAL (*n* = 3). *C*, expression of syntaxin isoforms 3, 4, and 7 was observed in TALs (*n* = 3).

Some SNAREs have been reported to be expressed in the TAL. They include VAMP2, VAMP3 (3), SNAP-23 (17), syntaxin 3, and syntaxin 4 (18–20). However, a more complete characterization of the SNARE expression profile is still missing. As a first step toward understanding the role of SNAREs in NKCC2 trafficking, we screened the expression of VAMP, SNAP, and syntaxin isoforms in TAL lysates (Fig. 1). In agreement with previous reports, we confirmed that VAMP2, VAMP3, SNAP-23, syntaxin 3, and syntaxin 4 are expressed in the TAL. In addition, we observed expression of VAMP7, VAMP8, SNAP-29, SNAP-47, and syntaxin 7. We did not detect expression of SNAP-25 in TALs.

##### Co-immunoprecipitation of NKCC2 and VAMP2 in Thick Ascending Limbs

**FIGURE 2.**
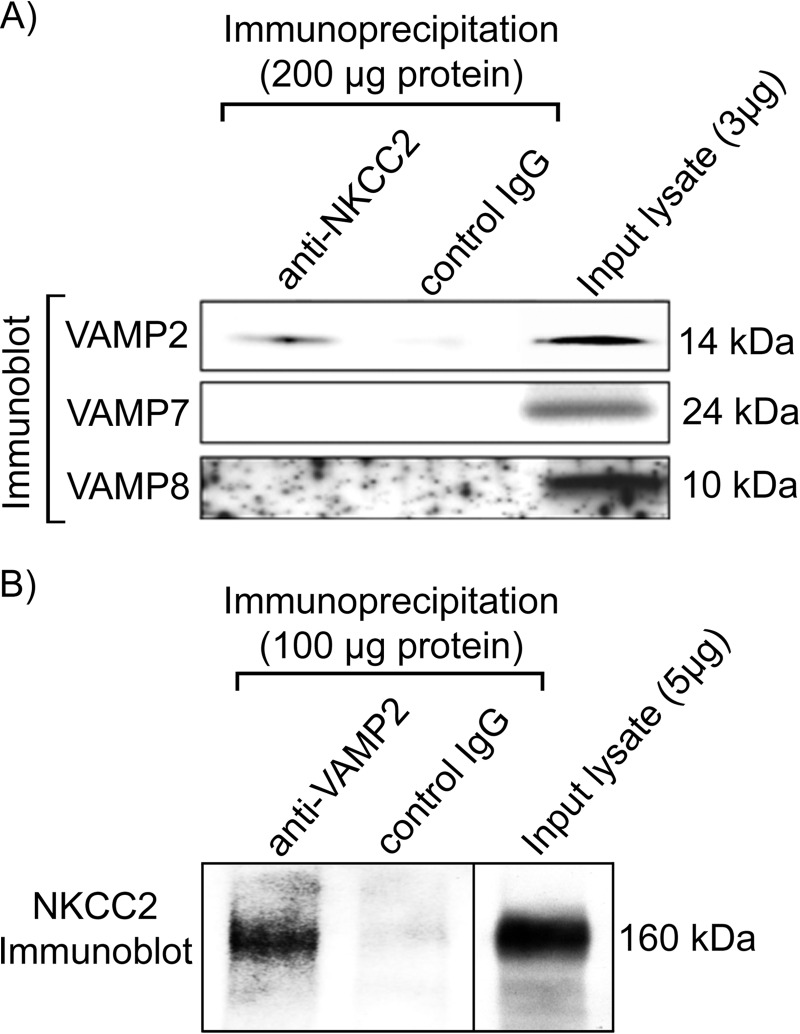
**VAMP2 co-immunoprecipitates with NKCC2.** *A*, co-immunoprecipitation between NKCC2 and different VAMPs revealed NKCC2 interaction (direct or indirect) with VAMP2 but not VAMP7 or VAMP8 in rat TAL lysates (200 μg of protein input; *n* = 4). *B*, reciprocal immunoprecipitation with an anti-VAMP2 antibody co-precipitated NKCC2 from TAL lysates (100 μg of protein input; *n* = 4). Control IgG failed to precipitate VAMP2/7/8 or NKCC2.

Previous data from our laboratory indicated that NKCC2 co-localizes with VAMP2 at both apical and subapical locations in TALs (3). To test whether NKCC2 may interact with VAMP2, we performed co-immunoprecipitation assays on fresh TAL lysates obtained from rats. We observed that VAMP2 co-immunoprecipitated with NKCC2 in TALs, whereas VAMP7 and VAMP8 did not (Fig. 2*A*). To confirm these results, we performed the reciprocal immunoprecipitation with an anti-VAMP2 antibody and detected NKCC2 by Western blot (Fig. 2*B*). Controls with non-immune IgG showed the absence of NKCC2 or VAMP2/7/8. These data indicate that VAMP2 interacts with NKCC2 or with a complex that contains NKCC2 in TALs.

**FIGURE 3.**
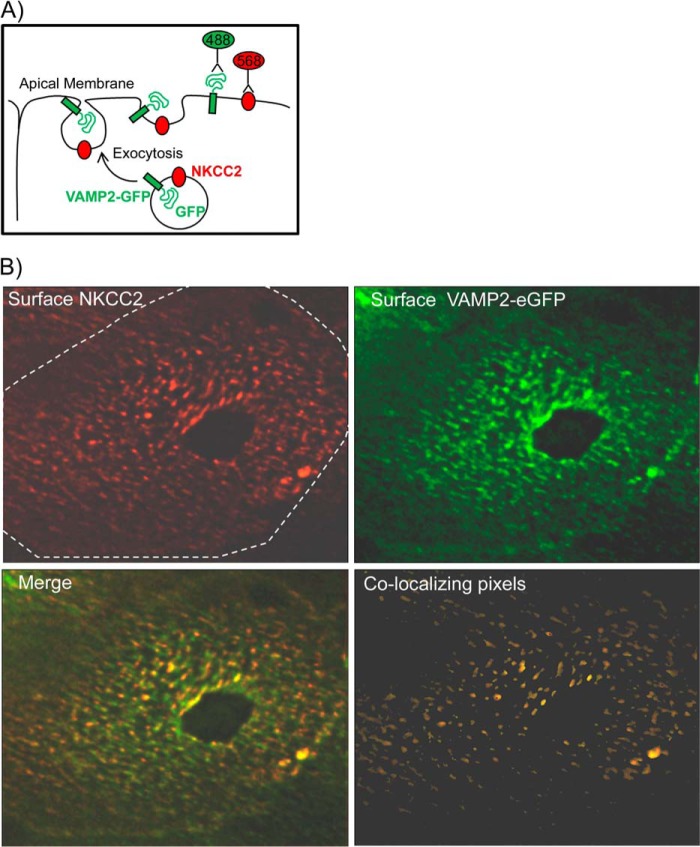
**NKCC2-VAMP2 co-localization at the apical surface of TALs.** *A*, diagram showing the exocytic process that allows immunodetection of surface NKCC2 and surface VAMP2-eGFP at the apical membrane of cultured TAL cells before fixation. *B*, apical distribution of surface NKCC2 (*red*) and surface VAMP2 (*green*) in cultured TALs. Apical surface NKCC2 was distributed in clusters. 45 ± 7% of NKCC2 clusters also contained VAMP2 (co-localizing pixels) (*n* = 5).

Once a vesicle fuses, VAMP2 remains inserted at the plasma membrane through its transmembrane domain. Because we found that VAMP2 interacted with NKCC2, we tested whether it co-localizes with NKCC2 at the apical surface of TALs. To label VAMP2 at the apical surface, we first generated polarized primary cultures of medullary TAL cells. TALs polarize within 4 days and express endogenous NKCC2. We then transduced TAL cells with a VAMP2 construct fused to eGFP in the carboxyl terminus (29), such that GFP faces the extracellular space when VAMP2 is in the plasma membrane (Fig. 3*A*). After cooling cells to 4 °C, apical surface VAMP2 was labeled with an anti-GFP antibody, and surface NKCC2 was labeled with an antibody against an exofacial epitope between TM5 and TM6 (7, 9). We observed that NKCC2 was distributed in a heterogeneous pattern restricted to clusters or discrete domains at the apical surface (Fig. 3*B*). VAMP2 localization was also heterogeneous, with most of the protein forming clusters. After image deconvolution and co-localization analysis, we determined that 45 ± 7% of NKCC2 clusters also contained VAMP2 (Fig. 3*B*). Taken together, our data indicate that VAMP2 interacts with NKCC2 and that they are located in similar microdomains at the apical surface of renal epithelial cells.

##### In Vivo Silencing of VAMP2 and VAMP3 in Thick Ascending Limbs

**FIGURE 4.**
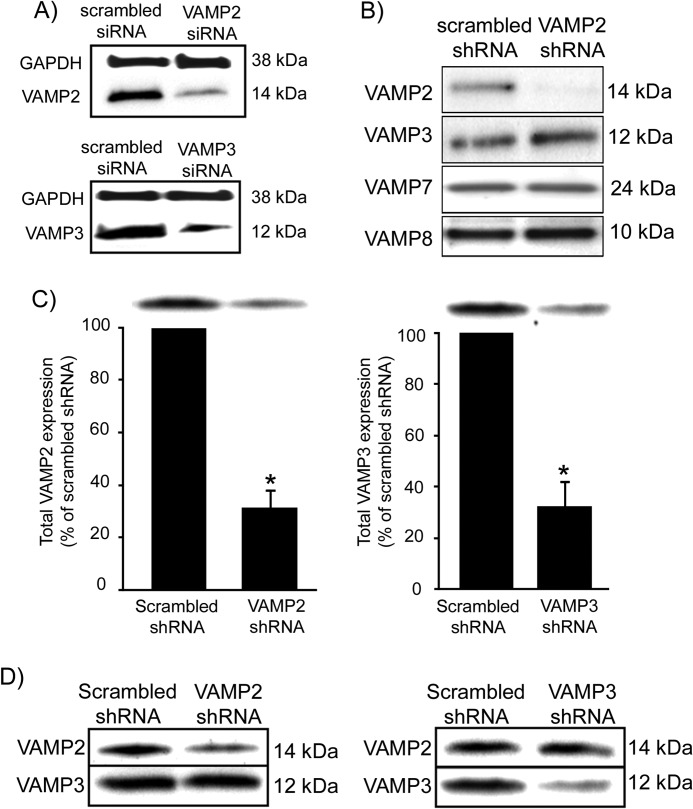
**Silencing of *VAMP2* and *VAMP3* in the renal medulla by *in vivo* gene transfer.** *A*, Western blot showing decreased VAMP2 and VAMP3 protein expression in NRK-52E cells after transfection with nude siRNAs. GAPDH expression was not affected (*n* = 3). *B*, Western blot showing VAMP2 protein expression in NRK-52E cells after transduction with adenoviruses carrying the corresponding shRNA. VAMP2-shRNA decreased VAMP2 expression but not VAMP3, VAMP7, or VAMP8 expression (*n* = 3). *C*, decrease in VAMP2 and VAMP3 protein expression after *in vivo* silencing in rat TALs. *VAMP2* silencing decreased VAMP2 expression by 69 ± 7% compared with control TALs from the same rat. *VAMP3* silencing decreased VAMP3 expression by 68 ± 10%. Values are expressed as mean percentage of scrambled shRNA, and *error bars* represent S.E. *, *p* < 0.01 *versus* scrambled shRNA (*n* = 3). *D*, representative Western blots from TALs isolated from rats injected with adenoviruses carrying VAMP2-shRNAs or VAMP3-shRNAs after 3 days of transduction. VAMP2-shRNA decreased VAMP2 but not VAMP3 expression and vice versa (*n* = 3).

We previously found that tetanus toxin blocked cAMP-stimulated NaCl transport in TALs (3), suggesting that tetanus toxin-sensitive VAMP isoforms (VAMP2/3) are involved in NKCC2 trafficking. Because tetanus toxin cleaves both VAMP2 and VAMP3, we developed a protocol to specifically decrease VAMP2 or VAMP3 expression in TALs via shRNA gene silencing. We designed siRNA sequences for *VAMP2* and *VAMP3*, targeting regions of the rat mRNAs that do not overlap with other VAMP isoforms. Then we synthesized nude siRNAs and tested their efficiency and selectivity by transfecting a rat kidney cell line (NRK-52E). Once silencing efficiency and specificity were confirmed by Western blot (Fig. 4*A*), we designed shRNA and produced adenovirus particles. The adenoviruses were tested again *in vitro* by transducing the rat cell line (Fig. 4*B*). We observed that expression of the targeted VAMP isoform was practically undetectable, whereas expression of other isoforms was unchanged. Next we used adenoviruses to deliver shRNAs to the outer medulla *in vivo* and determined the time course for maximal silencing in rat TALs. Expression of the target proteins was monitored at 2, 3, and 4 days in TAL suspensions obtained from the injected kidneys. We determined that VAMP2 and VAMP3 silencing was maximal at 72–96 h after injection, reaching a 69 ± 7% decrease for VAMP2 and 68 ± 10% for VAMP3 expression (*p* < 0.05) (Fig. 4*C*) compared with TALs from the contralateral kidney injected with scrambled shRNA adenoviruses (Fig. 4*D*). *VAMP2* silencing did not affect VAMP3 expression; nor did *VAMP3* silencing affect VAMP2 expression, indicating specificity of the targeted sequences *in vivo*.

##### Effect of VAMP2 and VAMP3 Silencing on cAMP-stimulated NKCC2 Trafficking

**FIGURE 5.**
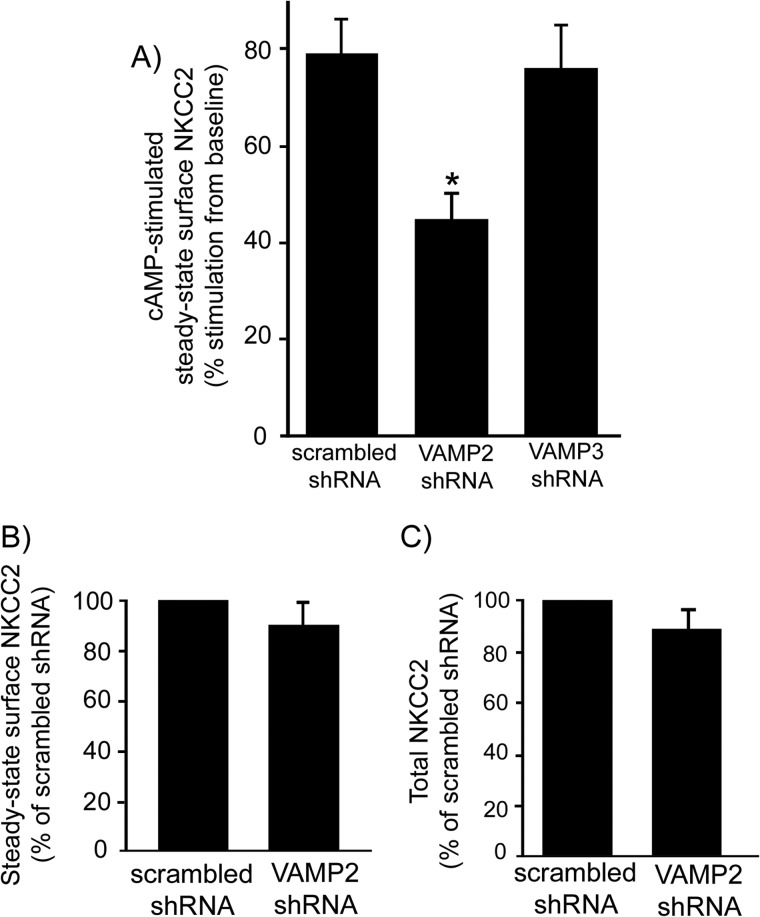
***VAMP2* silencing decreases cAMP-stimulated steady-state surface NKCC2 expression in rat TALs.** *A*, TALs were treated with forskolin (20 μm) plus IBMX (0.5 mm) for 30 min at 37 °C to stimulate intracellular cAMP production. Steady-state surface NKCC2 was measured by surface biotinylation in adenovirus-transduced TALs. *VAMP2* silencing decreased cAMP-stimulated steady-state surface NKCC2, but *VAMP3* silencing did not (scrambled, 79 ± 7% stimulation; VAMP2-shRNA, 45 ± 6% stimulation; VAMP3-shRNA, 76 ± 9% stimulation; *n* = 7). *B*, *VAMP2* silencing did not affect baseline constitutive steady-state surface NKCC2 (scrambled, 100%; VAMP2-shRNA, 91 ± 8%; *n* = 7). *C*, *VAMP2* silencing did not affect total NKCC2 protein expression (scrambled, 100%; VAMP2-shRNA, 86 ± 7%; *n* = 7). Values are expressed as mean percentage of scrambled shRNA. *Error bars*, S.E.; *, *p* < 0.05 *versus* scrambled shRNA.

To determine the role of VAMP2 and VAMP3 in cAMP-stimulated NKCC2 trafficking, we silenced *VAMP2* and *VAMP3 in vivo*. We measured steady-state surface NKCC2 in TAL suspensions after stimulating cAMP with forskolin + IBMX. In control TALs, cAMP stimulation increased steady-state surface NKCC2 by 79 ± 7% over baseline (*p* < 0.05) (Fig. 5*A*). In TALs where *VAMP2* was silenced, cAMP stimulated surface NKCC2 to only 45 ± 6% from baseline, representing a 43% blockade of the stimulatory effect of cAMP (*p* < 0.05). In contrast, silencing *VAMP3* did not affect cAMP-stimulated surface NKCC2, which rose to 76 ± 9% from baseline. Silencing *VAMP2* did not affect baseline steady-state surface NKCC2 expression (scrambled shRNA, 100% *versus* VAMP2-shRNA, 91 ± 8%) (Fig. 5*B*). Intracellular protein GAPDH was not detected at the surface fraction in surface biotinylation experiments (not shown). In addition, *VAMP2* silencing did not affect total NKCC2 expression (scrambled shRNA, 100% *versus* VAMP2-shRNA, 86 ± 7%) (Fig. 5*C*). These observations indicate that VAMP2 mediates cAMP-stimulated steady-state surface NKCC2 expression, whereas VAMP3 does not.

**FIGURE 6.**
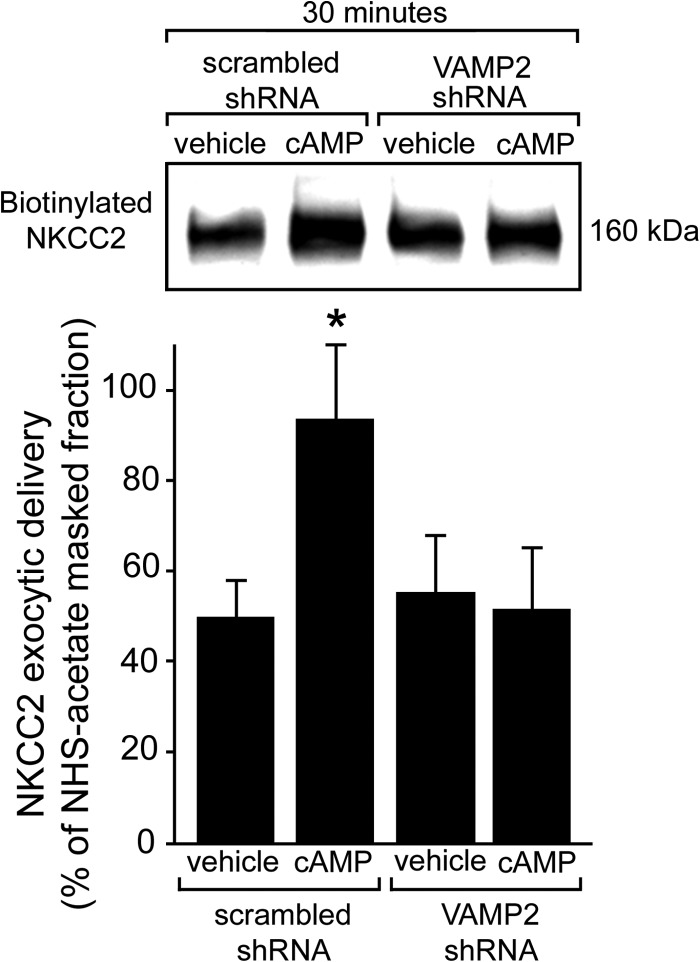
***VAMP2* silencing blocks cAMP-stimulated NKCC2 exocytic delivery in rat TALs.** Exocytic delivery of NKCC2 was measured as biotinylated NKCC2 at the TAL surface after masking with NHS-acetate (representative blot in the *top panel*). Constitutive NKCC2 exocytic delivery at 30 min was not affected by *VAMP2* silencing (scrambled, 49 ± 9%; VAMP2-shRNA, 55 ± 13%), but cAMP-stimulated NKCC2 exocytic delivery was completely blocked (scrambled, 93 ± 16%; VAMP2-shRNA, 51 ± 14%). Values are expressed as mean percentage of NHS-acetate-masked fraction. *Error bars*, S.E.; *, *p* < 0.05 *versus* vehicle/scrambled shRNA (*n* = 5).

Previously, we found that most of the stimulatory effect of cAMP on steady-state surface NKCC2 was mediated by increased rates of exocytic delivery to the apical surface (9). To determine whether VAMP2 mediates cAMP-stimulated exocytic delivery of NKCC2 to the cell surface, we silenced VAMP2 expression and measured exocytic delivery of NKCC2 over 30 min in TALs. In control TALs, baseline NKCC2 exocytic delivery over 30 min averaged 49 ± 9% of the total surface pool, and it was stimulated by cAMP to 93 ± 16%, a 90% increase (*p* < 0.05) (Fig. 6). In TALs transduced with VAMP2-shRNA, cAMP-stimulated NKCC2 exocytic delivery was completely blocked, whereas baseline NKCC2 delivery was not affected (baseline, 55 ± 13% *versus* VAMP2-shRNA, 51 ± 14%; *p* < 0.05). These data indicate that VAMP2 mediates all of the cAMP-stimulated NKCC2 exocytic insertion without affecting constitutive delivery of NKCC2 to the apical surface.

##### Effect of VAMP2 Silencing on NKCC2 Phosphorylation

**FIGURE 7.**
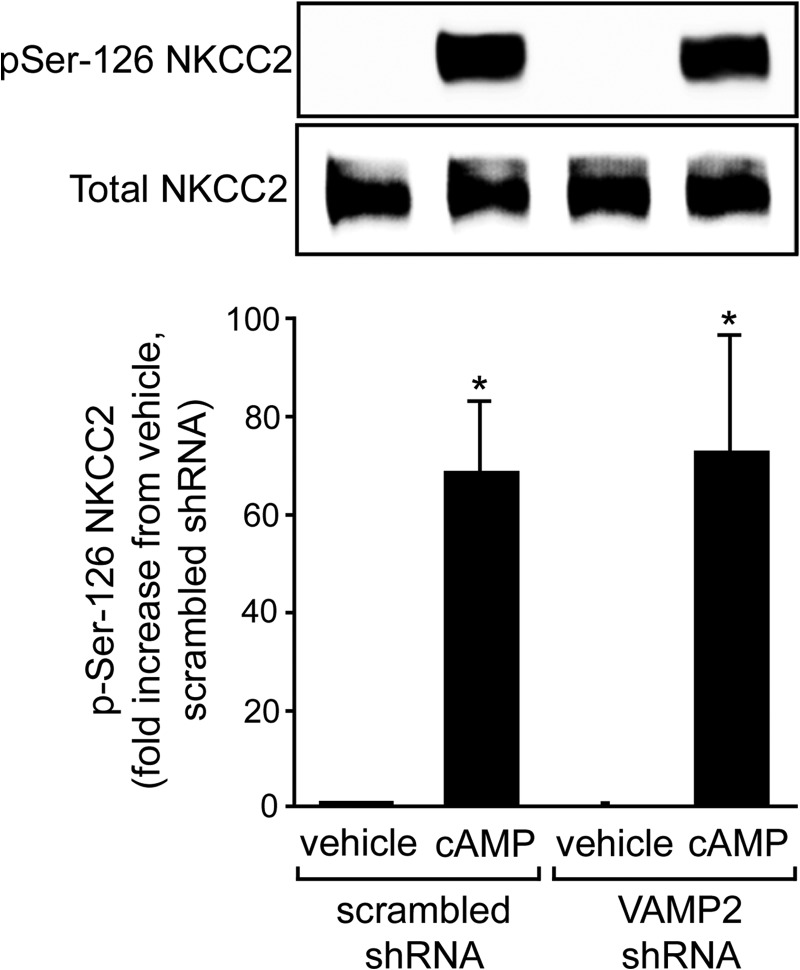
***VAMP2* silencing does not affect NKCC2 phosphorylation at Ser-126 in TALs.** NKCC2 phosphorylation at Ser-126 (*pSer-126*) was measured with an anti-phospho antibody. Baseline NKCC2 phosphorylation at Ser-126 was undetectable and was not affected by *VAMP2* silencing. Stimulation with 100 μm db-cAMP for 20 min enhanced NKCC2 Ser-126 phosphorylation 69 ± 14-fold in control TALs and 73 ± 24-fold in VAMP2-shRNA-transduced TALs. The degree of stimulation by db-cAMP was not statistically significant. Values are expressed as mean -fold increase from vehicle/scrambled shRNA, and *error bars* represent S.E. *, *p* < 0.05 *versus* vehicle (*n* = 4).

Our data indicate that VAMP2 mediates all of the cAMP-stimulated NKCC2 exocytic delivery and that VAMP2 interacts with NKCC2. Recent data indicate that cAMP induces phosphorylation of NKCC2 at Ser-126 within a PKA consensus site (28). Because we found that PKA mediates cAMP-stimulated NKCC2 exocytic delivery (9), it is possible that the inhibitory effect of *VAMP2* silencing we observed was due to decreased PKA-mediated Ser-126 phosphorylation. To test this, we measured cAMP-stimulated NKCC2 phosphorylation at Ser-126 with a phospho-specific antibody (28). We used the cAMP analog db-cAMP to better control cAMP levels. We found that db-cAMP (250 μm) enhanced NKCC2 Ser-126 phosphorylation to similar levels in control or VAMP2-shRNA-transduced TALs (Fig. 7). These data indicate that *VAMP2* silencing does not decrease the ability of PKA to phosphorylate NKCC2 and suggest that VAMP2 either interacts with NKCC2 downstream from NKCC2 phosphorylation or else is not part of the same pathway.

##### Effect of cAMP on VAMP2 Exocytic Delivery

**FIGURE 8.**
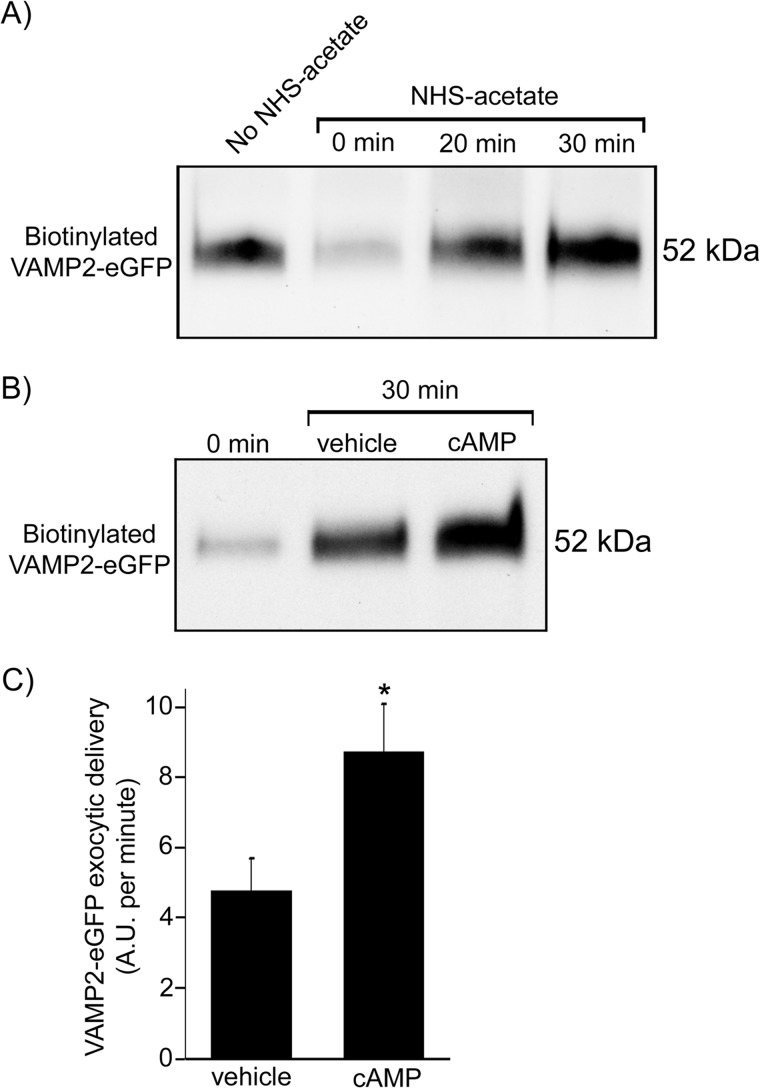
**cAMP stimulates exocytic delivery of VAMP2 to the TAL cell surface.** *A*, primary cultures of TALs were transfected with VAMP2-eGFP, and the time course of exocytic delivery was measured as biotinylated VAMP2-eGFP at the cell surface after masking with NHS-acetate. Biotinylated VAMP2-eGFP signal decreased on average by 78 ± 1% after masking with NHS-acetate (time 0), and it gradually recovered after 20 and 30 min at 37 °C. *B*, representative Western blot showing increase in VAMP2-eGFP exocytic delivery after stimulation of cAMP with forskolin (20 μm) plus IBMX (0.5 mm) for 30 min. *C*, cAMP stimulation enhanced the rate of VAMP2-eGFP delivery to the cell surface from 4.8 ± 0.9 to 8.7 ± 1.4 arbitrary units (*A.U.*)/min. Arbitrary units are defined as percentage of the NHS-acetate-masked fraction. *Error bars*, S.E.; *, *p* < 0.05 *versus* vehicle (*n* = 4).

We found VAMP2 at the apical membrane of TALs and found that VAMP2 mediates cAMP-stimulated NKCC2 exocytic delivery. Therefore, we next tested whether cAMP also stimulates delivery of VAMP2 to the apical surface. We transfected TAL primary cultures with the VAMP2-eGFP construct and measured the time course of VAMP2-eGFP exocytic delivery at 20 and 30 min by surface biotinylation (Fig. 8*A*). Masking of surface proteins with NHS-acetate decreased biotinylated VAMP2-eGFP signal by 78 ± 1%, similar to what we observed previously for NKCC2 (9). Stimulation of cAMP with forskolin + IBMX increased the rate of VAMP2-eGFP exocytic delivery by more than 80% (vehicle, 4.8 ± 0.9 arbitrary units/min *versus* cAMP, 8.7 ± 1.4 arbitrary units/min; *p* < 0.05) (Fig. 8, *B* and *C*). These results indicate that cAMP stimulates VAMP2 exocytosis in TALs.

**FIGURE 9.**
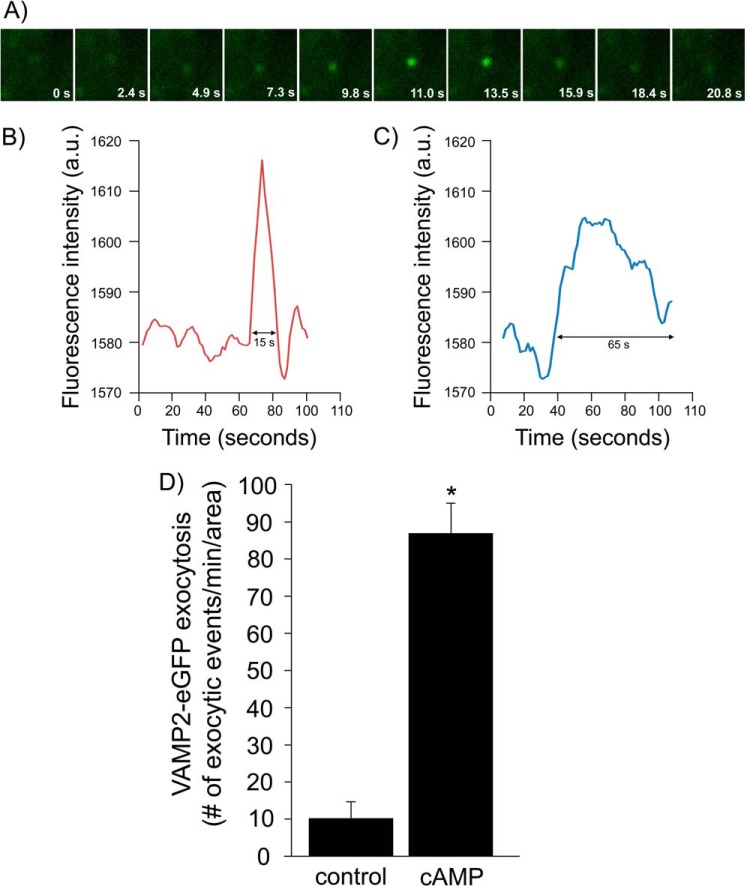
**cAMP stimulates exocytosis of individual VAMP2-carrying vesicles in renal epithelial cells.** *A*, representative time course of a real-time exocytic event in LLC-PK1 cells. Cultured cells were transfected with VAMP2-eGFP, and transient exocytic events were measured in real time by TIRF microscopy. *B*, representative trace of the change in fluorescence over time within a region of interest where an exocytic event shorter than 20 s occurred. *a.u.*, arbitrary units. *C*, representative trace of the change in fluorescence over time within a region of interest where a VAMP2-eGFP vesicle remained in close proximity to the membrane for a time lapse of at least 65 s before disappearing. *D*, quantification of the type of events described in *B*. Stimulation with cAMP increased the number of VAMP2-eGFP exocytic events from 10 ± 4 to 87 ± 8 events/min/area. Values are expressed as the number of exocytic events/min/area. *Error bars*, S.E.; *, *p* < 0.01 *versus* control (*n* = 3).

Because VAMP2 exocytic delivery to the plasma membrane measured by surface biotinylation is the result of many exocytic events, we monitored individual exocytic events for VAMP2 by TIRF microscopy. We transduced renal LLC-PK1 cells with VAMP2-eGFP adenoviruses and then imaged the region immediately adjacent to the plasma membrane. We observed a transient appearance of single VAMP2-carrying vesicles at the plasma membrane, evidenced by increased fluorescence intensity (Fig. 9*A*). We monitored the duration of those events and defined an exocytic event as one where the increase lasted less than 20 s, as shown in the representative trace in Fig. 9*B*. We also observed events in which the VAMP2-eGFP vesicle remained in close proximity to the plasma membrane for a period of time longer than 20 s (Fig. 9*C*). Those were not considered exocytic events. In control conditions, we detected 10 ± 4 events/min/area over a period of 20 min (Fig. 9*D*). When we added forskolin + IBMX to stimulate cAMP, we observed 87 ± 8 events/min/area, an 8.7-fold increase in the number of exocytic events (*p* < 0.01). These data indicate that cAMP stimulates exocytosis of VAMP2-containing vesicles in renal epithelial cells.

##### Effect of cAMP on VAMP2-NKCC2 Interaction

**FIGURE 10.**
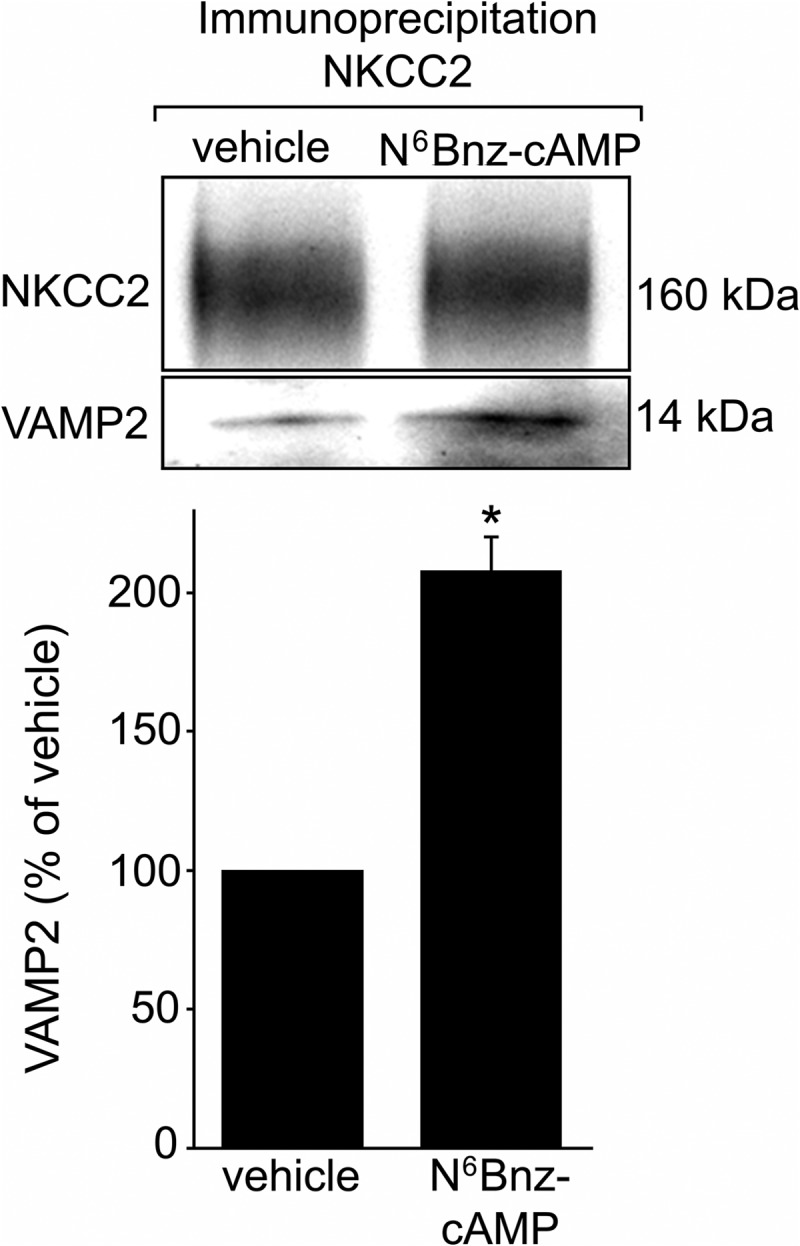
**Stimulation of PKA enhances VAMP2-NKCC2 interaction in TALs.** Incubation of TAL suspensions with a 1 mm concentration of the PKA-selective agonist *N*^6^Bnz-cAMP for 30 min enhanced NKCC2-VAMP2 interaction by 108 ± 12%, as measured by co-immunoprecipitation. Treatment with *N*^6^Bnz-cAMP did not affect NKCC2 protein levels (*top*). Values are expressed as mean percentage of vehicle, and *error bars* represent S.E. *, *p* < 0.01 *versus* vehicle (*n* = 5).

We have found previously that cAMP-stimulated NKCC2 exocytic delivery is mediated by PKA (9). Here we show that VAMP2 interacts with NKCC2, and silencing *VAMP2* completely blocks cAMP-stimulated NKCC2 exocytic delivery. We next tested whether stimulation of PKA enhances VAMP2-NKCC2 interaction. For this, we co-immunoprecipitated NKCC2 and VAMP2 from TALs treated with vehicle or the PKA agonist *N*^6^-benzoyl-cAMP + IBMX for 30 min. We found that PKA stimulation enhanced NKCC2-VAMP2 interaction, as demonstrated by an increase in the amount of VAMP2 pulled down by NKCC2 immunoprecipitation (Fig. 10). No interaction with non-immune IgG was detected, and no change in either total VAMP2 or NKCC2 expression was observed during treatment with the PKA agonist. These data suggest that the mechanism by which cAMP and PKA enhance NKCC2 exocytic delivery may involve increased interaction of VAMP2 with NKCC2.

## DISCUSSION

The abundance of transporters at the cell surface determines the rate of ion transport independently of the intrinsic activity of individual NKCC2 molecules. Thus, trafficking of NKCC2 to the apical membrane is an important mechanism for regulation of NaCl absorption by the TAL. The tight correlation between NKCC2 presence in the apical membrane and NaCl transport has been documented by us and others (3–8). Abnormally enhanced trafficking of NKCC2 to the apical membrane is related to salt-sensitive and salt-resistant hypertension (32, 33), underscoring the importance of this biological process in human disease.

The molecular mechanisms that regulate NKCC2 at the cell surface are poorly understood. Here we provide evidence that many members of the SNARE family of membrane fusion proteins are expressed in the TAL. We studied in particular VAMP2 and found that it specifically mediates cAMP-stimulated NKCC2 exocytic delivery into the apical membrane. We propose a model where VAMP2 is associated with NKCC2 in an intracellular pool of vesicles that functionally responds to cAMP stimulation. In non-polarized cells, VAMP2 has been linked to signaling-stimulated trafficking processes (21–24). In adipose cells, VAMP2 and VAMP3 are physically segregated in two different GLUT4-positive compartments (21). Moreover, in both adipose and muscle cells, insulin is only able to stimulate translocation of the VAMP2-associated GLUT4 pool (22, 29, 34, 35). Despite the importance of VAMPs and other SNAREs in human physiology, the specificity and role of VAMPs in renal function *in vivo* are poorly understood. In the particular case of VAMP2, addressing its biological role *in vivo* is difficult because deletion of the *VAMP2* gene is lethal *in utero. VAMP8* knock-out mice show defective cAMP-stimulated aquaporin-2 translocation in the collecting duct (36), a renal epithelium distal from TALs that expresses VAMP2, VAMP3, and VAMP8. However, in a collecting duct cell line, VAMP2 and VAMP3 were shown to be involved in constitutive and cAMP-stimulated aquaporin-2 translocation (37). Thus, the specific role of VAMP2 in exocytosis in renal epithelial cells is still not clear. In specialized secretory cells of the kidney termed juxtaglomerular cells, cAMP-stimulated exocytosis is specifically mediated by VAMP2 but not VAMP3 (26). The evidence we present here constitutes the first indication that VAMP2 interacts with a NaCl co-transporter to mediate cAMP-stimulated apical exocytic trafficking in native renal epithelial cells.

Our data show that silencing *VAMP2* by ∼70% blocked cAMP-stimulated steady-state surface NKCC2 by 50% and completely blocked the cAMP-stimulated NKCC2 exocytic delivery. These data are intriguing and suggest that (i) the effectiveness of the silencing procedure was adequate, because blockade of exocytic delivery was complete, and (ii) the partial blockade of stimulated steady-state surface NKCC2 was not due to ineffective silencing. This partial blockade of steady-state surface NKCC2 during complete blockade of exocytic delivery may reflect retention of NKCC2 at the surface due to decreased endocytosis. We recently demonstrated that NKCC2 undergoes constitutive endocytosis mediated by dynamin 2, clathrin, and lipid rafts (10). Inhibition of these processes induced retention of NKCC2 at the plasma membrane and increased steady-state surface NKCC2 (8, 10). Although our data show that most cAMP-stimulated surface NKCC2 is mediated by enhanced exocytic delivery mediated by VAMP2, we cannot rule out the possibility that a fraction of the increase in steady-state surface NKCC2 caused by cAMP is due to decreased endocytosis.

We also found that NKCC2 co-immunoprecipitates with VAMP2 in the TAL. The experimental design used here does not allow us to determine whether this interaction is direct or via intermediate proteins forming part of a multicomponent complex. However, the functional significance is emphasized by the observation that *VAMP2* silencing prevents cAMP-stimulated NKCC2 exocytic delivery. Although we think this is the first report indicating interaction of a SNARE protein with an apical sodium transporter, in neurons, VAMP2 physically interacts with P/Q-type calcium channels (38) and Kv2.1 potassium channels (39, 40), modulating channel activity. Interestingly, we describe for the first time that NKCC2 is located in apical surface clusters, as imaged by confocal microscopy using exofacial antibodies to label NKCC2 in primary cultures of TAL cells. Although the biological significance of this membrane compartmentalization is not clear, in neurons, membrane clusters composed of non-conducting ion channels serve as platforms for exocytosis (41, 42). We also found that a fraction (∼45%) of these NKCC2 domains also contained VAMP2-eGFP. This novel observation opens the possibility that VAMP2-mediated exocytosis and delivery of NKCC2 may be more likely to occur at defined domains at the cell surface. The mechanism by which this may occur is not clear from the present work, but it may provide new directions for studying the regulation of renal transporters.

Hormonal stimulation of cAMP is a potent stimulus for NKCC2-mediated NaCl transport in the TAL. Most cAMP stimulation of NKCC2 exocytic delivery is mediated by PKA (9). We found that, despite completely blocking NKCC2 delivery, silencing *VAMP2* did not affect NKCC2 phosphorylation at Ser-126, which is a target of PKA. Thus, our data indicate that *VAMP2* silencing does not affect PKA activity and that VAMP2 is downstream from NKCC2 phosphorylation in the signaling cascade leading to NKCC2 exocytic delivery. Our data may also suggest that Ser-126 phosphorylation is not sufficient for cAMP to stimulate NKCC2 exocytosis. Whether VAMP2 or other SNAREs are targets of PKA-mediated phosphorylation in the TAL is unclear. In parotid acinar cells, the VAMP2-dependent stimulatory effect of PKA on granule exocytosis is mediated by a non-identified intermediate protein (43). We know of no data suggesting that VAMP2 may be a target of PKA. However, many regulatory proteins that modulate SNARE function, including snapin (44), tomosyn (45), complexin (46), and Munc18 (47), are targets of the PKA pathway. Currently, we cannot rule out the possibility that PKA-mediated phosphorylation of SNARE accessory proteins is involved in VAMP2-mediated NKCC2 trafficking.

We previously showed that cAMP stimulates surface NKCC2 by enhancing exocytic delivery via PKA (9). Here, we show that VAMP2 mediates all of the stimulatory effect of cAMP on NKCC2 exocytosis (Fig. 5) and that activation of PKA enhances the NKCC2-VAMP2 interaction, as measured by co-immunoprecipitation (Fig. 10). These data identify VAMP2 as a component of the cAMP-stimulated exocytic machinery for NKCC2. However, it is still unclear how VAMP2 mediates NKCC2 exocytosis and whether VAMP2 interaction with NKCC2 is required for cAMP-stimulated NKCC2 exocytosis. We propose that this is a step following PKA activation, required to enhance the NKCC2 exocytic rate. It is possible that NKCC2 and VAMP2 reside in the same vesicles, as shown for other membrane transporters (21, 48). We speculate that PKA stimulation enhances the interaction of NKCC2 and VAMP2 by at least three possible mechanisms as follows. (i) Phosphorylation of NKCC2 recruits VAMP2 and other components of the exocytic machinery to NKCC2- and VAMP2-containing vesicles in response to PKA. In the TAL, PKA directly phosphorylates NKCC2 (28), but the role of this event in exocytosis or in promoting protein-protein interactions is unclear. (ii) Phosphorylation of VAMP2 or a VAMP2-interacting SNARE may promote the assembly of a multiprotein complex required for NKCC2 exocytosis. In other cells, PKA-mediated phosphorylation stimulates the assembly of the SNARE complex. NKCC2 may be part of this exocytic complex in TALs. In neurons, calcium channels interact with different SNAREs, and their phosphorylation regulates these interactions (49, 50). (iii) SNARE accessory proteins like tomosyn and snapin are phosphorylated by PKA, resulting in increased exocytosis (44, 45). Whether NKCC2 interacts with any SNARE accessory protein is unknown. However, there is evidence that snapin interacts with other renal apical transporters (UT-A1 urea transporter) (51, 52). Alternatively, VAMP2 may interact with NKCC2 in a chaperone-like manner independently of its role in exocytosis. This kind of interaction has previously been reported for potassium (53–56) and calcium channels (57–59) in other cells. This may occur at the vesicle or at the surface after NKCC2 exocytosis has occurred. We found that NKCC2 and VAMP2 co-localize at the apical surface of TALs (Fig. 3). However, it is unknown whether VAMP2 serves as a regulatory protein for NKCC2 activity once at the cell surface. We consider that further investigation into these mechanisms may provide information on how VAMP2 mediates cAMP-stimulated NKCC2 exocytosis. In conclusion, we consider that PKA stimulation of NKCC2-VAMP2 interaction is central to enhanced exocytosis, but the precise molecular mechanism is unknown.

Overall, we have characterized a VAMP2-mediated pathway in renal epithelial cells that specifically mediates cAMP-stimulated NKCC2 exocytic delivery. VAMP2 does not seem to play a major role in constitutive NKCC2 delivery. We have also demonstrated that NKCC2 physically interacts with VAMP2 in the TAL and that they co-localize at the apical surface of TAL cells. Activation of PKA enhanced VAMP2-NKCC2 interaction, which may be part of the mechanism by which cAMP stimulates NKCC2.

## Footnotes

2 The abbreviations used are:

TAL: thick ascending limb
IBMX: 3-isobutyl-1-methylxanthine
*N*^6^-Bnz-cAMP: *N*^6^-benzoyl-cAMP
db-cAMP: dibutyryl-cAMP
NHS: *N*-hydroxysulfosuccinimide
NSF: *N*-ethylmaleimide-sensitive factor
VAMP: vesicle-associated membrane protein
SNAP: synaptosome-associated protein
eGFP: enhanced GFP
TIRF: total internal reflection fluorescence.
